# Evaluation of standardized performance test methods for biomedical Raman spectroscopy

**DOI:** 10.1117/1.JBO.27.7.074705

**Published:** 2021-10-28

**Authors:** Andrew M. Fales, Ilko K. Ilev, T. Joshua Pfefer

**Affiliations:** U.S. Food and Drug Administration, Center for Devices and Radiological Health, Silver Spring, Maryland, United States

**Keywords:** Raman spectroscopy, standards, test methods, turbid phantom

## Abstract

**Significance:**

Raman spectroscopy has emerged as a promising technique for a variety of biomedical applications. The unique ability to provide molecular specific information offers insight to the underlying biochemical changes that result in disease states such as cancer. However, one of the hurdles to successful clinical translation is a lack of international standards for calibration and performance assessment of modern Raman systems used to interrogate biological tissue.

**Aim:**

To facilitate progress in the clinical translation of Raman-based devices and assist the scientific community in reaching a consensus regarding best practices for performance testing.

**Approach:**

We reviewed the current literature and available standards documents to identify methods commonly used for bench testing of Raman devices (e.g., relative intensity correction, wavenumber calibration, noise, resolution, and sensitivity). Additionally, a novel 3D-printed turbid phantom was used to assess depth sensitivity. These approaches were implemented on three fiberoptic-probe-based Raman systems with different technical specifications.

**Results:**

While traditional approaches demonstrated fundamental differences due to detectors, spectrometers, and data processing routines, results from the turbid phantom illustrated the impact of illumination-collection geometry on measurement quality.

**Conclusions:**

Specifications alone are necessary but not sufficient to predict *in vivo* performance, highlighting the need for phantom-based test methods in the standardized evaluation of Raman devices.

## 1 Introduction

Due to the unique, molecular-specific information provided by Raman spectroscopy, it has great potential for use in the study of various diseases. Raman-based diagnostics can be developed by monitoring the biochemical changes that occur during disease progression. Cancer detection is the most widely studied application of Raman technology, with devices for skin cancer detection and brain tumor surgical guidance currently being developed for commercialization.^1^ Non-invasive glucose monitoring is another active area of research, with at least one product in development.^2^^,^^3^ Other applications include cardiovascular, inflammatory, and retinal diseases.^4^^,^^5^ While these devices have delivered promising clinical results, there are currently no legally marketed Raman-based diagnostic medical devices in the United States and such devices will need to undergo the appropriate premarket regulatory review. The wide range of instrument configurations, measurement protocols, and data processing techniques can complicate the regulatory review process and slow the arrival of this novel technology to the market.

The medical device industry often relies on the use of consensus standards to assess fundamental performance and demonstrate that products meet specifications. In doing so, the complexity of the premarket regulatory review process can be reduced, providing more consistency, and resulting in faster time-to-market. Standards can also help to facilitate early device development, enable inter-device comparison in the literature, and enable postmarket consistency (quality assurance testing and recalibration). Consensus standards are developed by national and international entities called Standards Developing Organizations (SDOs). A few examples of SDOs include the International Organization for Standardization (ISO), the American National Standards Institute (ANSI), and ASTM International, formerly, the American Society for Testing and Materials. In the case of Raman spectroscopy, there exist some general standards related to performance testing, calibration, and relative intensity correction of a Raman spectrometer (ASTM E1683, E1840, E2529, E2911). Although these standards are specific to the spectrometer itself, they are applicable to all spectrometer-based Raman optical delivery approaches, including fiber-optic, free-space, and microscopy systems.^6^^–^^8^ The only standard to address the use of Raman spectroscopy for an application is in the analysis of liquefied natural gas (ASTM D7940).

While these standards outline some of the key performance characteristics of Raman spectrometers, they do not address the various design considerations that are important in the development of a diagnostic instrument. Some of these factors include fiber optic probe design, spectral pre-processing (background/fluorescence subtraction, smoothing, normalization, etc.), and diagnostic algorithm. Well-established medical imaging modalities, such as x-ray computed tomography, magnetic resonance imaging, and ultrasound regularly use tissue-simulating phantoms to assist in the performance evaluation of devices that can differ greatly in their components and processing methods from one manufacturer to the next. Phantoms are objects used to mimic the relevant properties of human tissues, specific to a medical imaging modality, to ensure that systems and methods are operating correctly.^9^ To date, most commonly used phantoms in Raman spectroscopy can be designed to mimic the optical absorption and scattering of tissue, but do not exhibit the Raman spectral components associated with the intended tissue type.^10^^–^^13^ Our laboratory has recently reported on a 3D-printed phantom based on polyacrylate resins with biologically relevant absorption and scattering properties, as well as a Raman spectrum similar to that of tissue.^14^ By varying the depth, size, and concentration of inclusions within phantoms, one can develop a robust set of test methods to characterize the performance of a complete Raman system.

There is an outstanding need for standardized approaches in the evaluation of emerging Raman diagnostic devices. Manufacturers of Raman instrumentation often advertise the higher throughput and/or sensitivity of their products over competitors; however, these claims are difficult to verify without a side-by-side comparison of both products. In addition to hardware performance, Raman diagnostics generally rely on the use of spectral processing algorithms. There are currently no standardized methods available for the combined evaluation of Raman diagnostic hardware and software. The purpose of this work is to evaluate current methods for Raman device performance characterization and investigate potential tools and methods to aid in benchmarking the performance of complete Raman systems.

In recent years, coordinated efforts have been made to investigate the cross-laboratory variability in Raman spectra. Itoh et al.^15^ compared the consistency of Raman shifts from spectra of polystyrene, benzonitrile, and cyclohexane obtained with 26 different systems.^15^ The use of three standards allowed the authors to conclude that deviations in wavenumber are due to the Raman systems rather than the material used. They observed poor consistency of Raman shifts, which may be due to the common use of the $520\;{\mathrm{cm}}^{-1}$ Si peak for offset correction. The authors note a nonuniform improvement in Raman shift consistency across the collected wavenumber range, with greater improvement closer to the Raman shift value used for offset correction. However, each instrument underwent calibration according to its manufacturer’s protocol, which may introduce an additional source of error. Another large-scale study by Guo et al.^16^ compared peak shifts (acetaminophen, polystyrene, cyclohexane), intensity variations, peak widths, and noise levels of 35 different Raman devices from 15 different institutions. Each instrument was calibrated using the same method, with acetaminophen as the wavenumber standard and a third-order polynomial calibration function. This study analyzed the cross-setup comparability and variability of the Raman systems and concluded that computational methods that can be shared across the community are urgently needed to remove the setup-induced variability. They recommend that Raman manufacturers include open access spectral calibration methods by-default and provide user access to raw data and encourage manufacturers to work together with researchers to develop standard procedures for verifying instrument calibration and performance. While these studies quantified the extent of inter-device variability of Raman spectra, further efforts are needed to fully investigate the sources of variability, one being the calibration method, and the impact of this variability on devices intended for *in vivo* clinical measurements.

In this report, we review the recommendations and specifications contained within current standards documents regarding performance evaluation of Raman spectrometers. These methods are then implemented on three different Raman systems to investigate the effect of various design parameters on device performance. We also demonstrate the use of a turbid phantom as a potential tool for the assessment of Raman diagnostic devices and show that knowledge of the technical specifications of a Raman system alone are likely not sufficient to predict effectiveness when performing measurements in biological tissue. The findings of this review and testing are discussed in terms of current challenges and obstacles in the development of Raman-based medical products. Our results indicate the need for additional consensus standards and guidelines in the biomedical Raman field to assist with clinical translation and regulatory review of this promising technology. The data presented here provide support for the methods proposed in existing standards and lay the groundwork for development of future standards to address the performance evaluation of Raman diagnostic devices.

## 2 Review of Consensus Standards

There are several standards related to Raman instrumentation that have been published by ASTM International. One of these is a standard practice, a definitive set of instructions for performing one or more specific operations that does not produce a test result. While the others are standard guides, a compendium of information or series of options that does not recommend a specific course of action. A summary of these standards is provided in Table 1.

**Table 1:** Summary of characteristics addressed in existing Raman performance standards.

	ASTM E1683^17^	ASTM E1840^18^	ASTM E2911^19^	ASTM E2529^20^
Latest Update	2014	2013	2013	2014
Document type	Practice (overview)	Guide (specific)	Guide (specific)	Guide (specific)
Raman shift calibration	x	x	—	—
Resolution	x	—	—	x
Stray light	x	—	—	—
Dark signal	x	—	—	—
Sensitivity	x	—	—	—
Relative intensity correction	—	—	x	—
Reference materials	• Carbon tetrachloride	• Naphthalene	• NIST SRM 2241 (785 nm), 2242 (532 nm), 2243 (488/514.5 nm), 2244 (1064 nm), 2245 (632.8 nm, 2246 (830 nm)	• Calcite
		• Bis-MSB		• Pen lamp
		• Sulfur		
		• Toluene/acetonitrile		
	• Cyclohexane			
		• Acetaminophen		
		• Benzonitrile		
	• Indene	• Cyclohexane		
	• Pen lamp	• Polystyrene		

### 2.1 ASTM E1683—Standard Practice for Testing the Performance of Scanning Raman Spectrometers

As a standard practice, ASTM E1683 provides a general description of the main performance characteristics relevant to Raman spectrometers.^17^ This practice was originally approved in 1995, prior to the widespread availability of multichannel detectors and as such, includes some outdated information that is specific only to scanning Raman spectrometers. For calibration, the relevant methods include spectral response correction and wavenumber calibration. While not specifically referenced, a standard guide for relative intensity correction using a NIST Standard Reference Material (ASTM E2911) is available.^19^ The provided references for wavenumber calibration have also been incorporated into a standard guide of Raman shift standards (ASTM E1840).^18^

Performance evaluation is divided into two main components, the monochromator and the detector. Relevant performance characteristics of the monochromator include resolution and stray light rejection. Methods for determining the resolution of a Raman spectrometer can be found in the standard guide ASTM E2529, which is covered below.^20^ Two ways of measuring stray light are described, both involving signal collection in the low wavenumber region ($<50\;{\mathrm{cm}}^{-1}$). While these methods were appropriate for the scanning spectrometers at the time, current multichannel instruments rely on filters to reject backscattering of the excitation light, which generally block all light below $∼300\;{\mathrm{cm}}^{-1}$. In this case, stray light can be investigated with filter-based methods, such as those found in the literature and ASTM E387, a standard test method for estimating stray radiant power of dispersive spectrophotometers.^21^^,^^22^

The detector performance characteristics, dark signal level and sensitivity, are outlined in reference to a photomultiplier tube (PMT). This represents another outdated part of the standard, as multichannel CCD detectors have become ubiquitous in the market. Although the standard refers to PMTs, the same performance characteristics apply to CCDs. The recommendations include regularly checking the dark signal level and measured signal intensity of the detector to ensure there is no degradation in performance. In practice, this would involve recording a dark spectrum and measurement of a reference sample before each use.

### 2.2 ASTM E1840—Standard Guide for Raman Shift Standards for Spectrometer Calibration

This guide includes Raman shift values for eight common liquid and solid materials that can be used for wavenumber calibration of Raman spectrometers. The shift values were independently determined by eight different laboratories, and only those peaks with a standard deviation of $<1\;{\mathrm{cm}}^{-1}$ were reported. The guide does not provide any methods for performing the actual calibration of the spectrometer. While the most common method for spectrometer calibration involves the use of atomic emission lines from low-pressure discharge lamps (mercury, argon, and neon), these lamps can present challenges when used with Raman instruments, especially when the exact laser wavelength is not known. The laser diodes that are often used in modern Raman systems generally have a center wavelength tolerance of ±0.5  nm. If the spectrometer is calibrated using the lamp emission lines and nominal laser wavelength, inconsistencies will arise from device-to-device. Using Raman shift standards, excited with the instrument’s laser, one no longer needs to know or determine the exact wavelength. Of the eight materials given in this guide, 4-acetamidophenol (Tylenol) is one of the most widely used Raman shift standards in the literature.

### 2.3 ASTM E2911—Standard Guide for Relative Intensity Correction of Raman Spectrometers

This standard guide outlines the use of NIST Standard Reference Materials (SRMs) in the 224X series to correct a Raman spectrometer for its relative intensity response function. Due to variations in the optical throughput and sensitivity of different instruments, the collected Raman spectra can show significant discrepancies in relative peak intensities. This is especially evident when different excitation wavelengths are used. To facilitate comparison of spectra between instruments, a standardized reference material with known spectral properties can be used. Spectral response calibration of a spectrometer is generally performed using a traceable calibrated irradiance source. However, in practice, it is difficult to properly position these lamps to reproduce the Raman sampling geometry. This, along with other challenges presented using an additional piece of equipment, led to the development of alternative methods using luminescence standards.

Luminescent glass materials designed and calibrated at NIST have been developed for excitation laser wavelengths of 785, 532, 488/514.5, 1064, 632.8, and 830 nm (SRM 2241, SRM 2242, SRM 2243, SRM 2244, SRM 2245, and SRM 2246, respectively). These SRMs allow a Raman instrument to be calibrated using the Raman excitation laser and can be placed in the same position as the sample. Unlike irradiance sources, these luminescent glass SRMs do not require periodic recalibration, owing to their high photostability. Each SRM has an associated certified polynomial that describes the known luminescence spectrum. By comparing the measured spectrum to the known output, a relative intensity correction function can be determined. Validation of the relative intensity correction can be performed by determining the ratios of chosen band areas of a measured Raman spectrum; approximate values are provided for cyclohexane.

The standard also provides some common issues that can be encountered while correcting Raman spectra. The first is polarization biases, which primarily arise from the polarization dependence of diffraction grating efficiency. Since the calibration source is unpolarized, the detection of polarized scattering can impact the relative peak intensities. The second is the positioning dependence of the calibration standard, where slight translations along the optical axis can alter the shape of the luminescence spectrum. If the SRM cannot be reliably positioned at the focus of the instrument, another location, where minor translations do not significantly impact the relative intensity of the measured luminescence spectrum should be used. Spectral resolution can also impact the relative intensity of some Raman bands, depending on their linewidths and overlap with nearby bands. Guide E2529 is referenced for additional information concerning spectral resolution.^20^ The final issue discussed is the effect of signal, noise and background. If the intensity calibration data are not collected with a high signal-to-noise ratio (SNR), noise propagation issues can be intensified in corrected spectra. The intensity calibration data should be collected with a high level of detector signal while avoiding saturation.

### 2.4 ASTM E2529—Standard Guide for Testing the Resolution of a Raman Spectrometer

Two methods for testing the resolution of a Raman spectrometer are presented in this guide. One using the emission lines of a low-pressure arc lamp, and the other using a calibrated Raman band of calcite. While gas discharge lamps are often used for testing the resolution of a spectrometer, this can have specific disadvantages for a Raman spectrometer. As previously noted, the sampling configuration of Raman devices often makes it difficult to properly align a lamp with the sample position, which could lead to distortion of the line shape. Another disadvantage is that the excitation laser line width can significantly broaden Raman spectral features. Thus, an ideal method would use a Raman-active material, excited by the device’s own laser, to provide peaks for resolution measurements. Calcite is presented as a chemically inert, stable, and safe material that can be used for such a purpose. Unfortunately, the calcite spectrum only contains a single well-characterized peak at $1085\;{\mathrm{cm}}^{-1}$. In theory, the resolution of a Raman spectrometer will increase with the Stokes shift due to the inverse relation between wavelength and wavenumber. However, most spectrometer designs exhibit focal plane curvature which results in the highest resolution being located at the center of the spectral range. For fixed-grating spectrographs, the $1085\;{\mathrm{cm}}^{-1}$ peak of calcite will typically fall near the center of the focal plane, providing a reasonable estimate for the system resolution.

## 3 Experimental

### 3.1 Materials and Instrumentation

Three different Raman spectrometers, one portable and two benchtop models, were employed in this study. The portable spectrometer (Device 1) was obtained from Ocean Optics (QEPro; Largo, Florida) and configured with the H4 600  g/mm grating, blazed at 750 nm, and a 100-μm slit. The second system (Device 2) consisted of a Princeton Instruments CCD detector and Acton spectrograph (InSight:400B, Newton, New Jersey) with a 600  g/mm grating blazed at 750 nm. The third system (Device 3) included an Andor CCD (iVac 316; Belfast, Northern Ireland) and lens-based spectrograph from EmVision (HT Spectrometer; Loxahatchee, Florida), containing a volume phase holographic transmission grating and 100-μm entrance slit. The EmVision spectrometer also includes a long-pass filter with an edge wavelength of ∼805  nm to reduce the amount of Rayleigh scatter reaching the detector. Excitation was provided by a wavelength-stabilized 785-nm laser from Innovative Photonics Solutions (I0785MM0350MF; Monmouth Junction, New Jersey). A fiber-coupled Raman probe from InPhotonics (RPB-785; Norwood, Massachusetts) was used to deliver excitation light to (105-μm excitation fiber) and collect (200-μm collection fiber) Raman scattering from the sample. This probe has a shared excitation and collection path with a focal length of 7.5 mm.

Wavelength calibration was performed using a mercury-argon lamp (HG-1) from Ocean Optics. Acetaminophen (A7085, Millipore Sigma; St. Louis, Missouri) was used for wavenumber calibration with known peak positions from ASTM E1840.^18^ A standard reference material (SRM-2241) was purchased from the National Institute of Standards and Technology (Gaithersburg, Maryland) for relative intensity correction of the three Raman systems. Stray light was characterized using a stabilized Tungsten-Halogen lamp (SLS201L, Thorlabs; Newton, New Jersey) and 850-nm long-pass filter (FEL0850, Thorlabs). Gold nanorods with a surface-enhanced Raman scattering (SERS) label (Ramanprobes™ with SERS label B, Part #C17-785-B-PEG-50, Nanopartz Inc., Loveland, Colorado) were purchased and diluted in deionized water to an optical density (OD) of 1 before use.

A tissue phantom was prepared using a triple-jetting 3D printer (Obet260 Connex3, Stratasys Ltd.; Eden Prairie, Minnesota) that cures liquid photopolymer with UV light. This printer has an X and Y axis resolution of 600 dpi, and a Z axis resolution of 1600 dpi, with an accuracy of 100  μm. The phantom was designed in SketchUp Free (Trimble Inc.; Sunnyvale, California) and printed using VeroWhitePlus resin (Stratasys Ltd.).

**Fig. 1:**
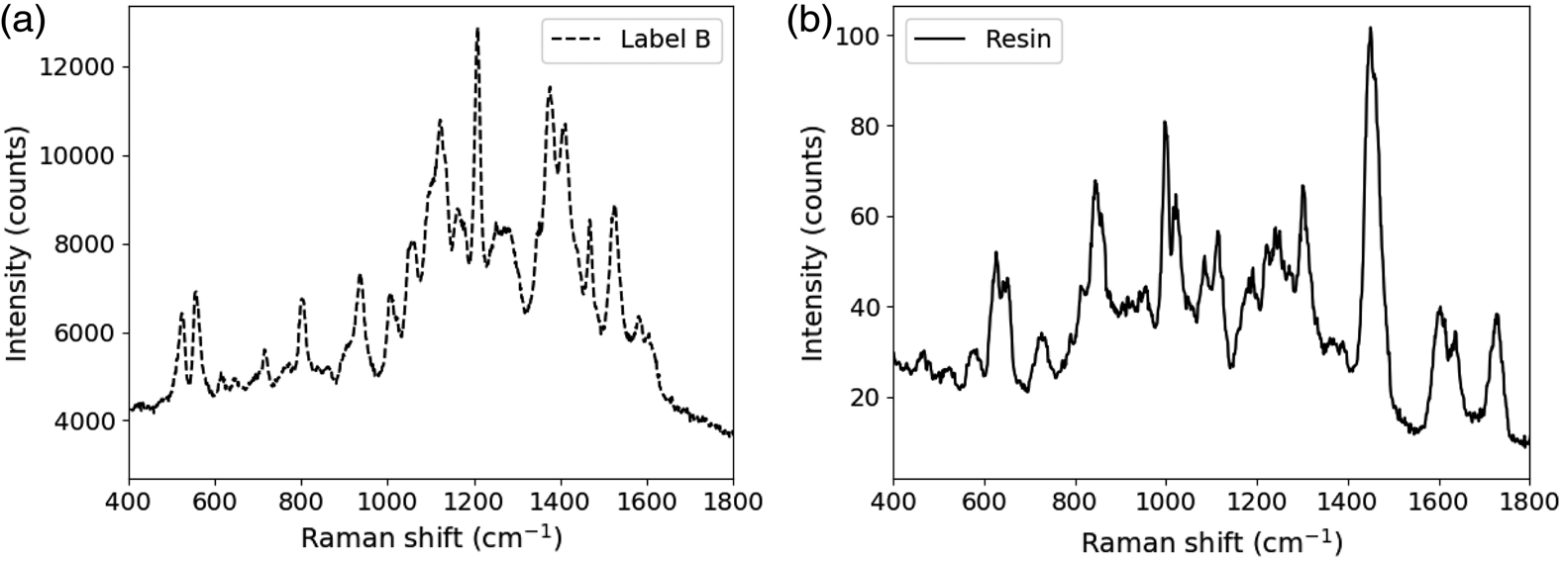
Raman spectra of (a) the gold nanorods and (b) printer resin material. Spectra were collected using Device 2 with a 100-ms integration time.

Representative Raman spectra of the Label B nanorods and printer resin are show in Fig. 1, below.

### 3.2 Phantom Measurements

**Fig. 2:**
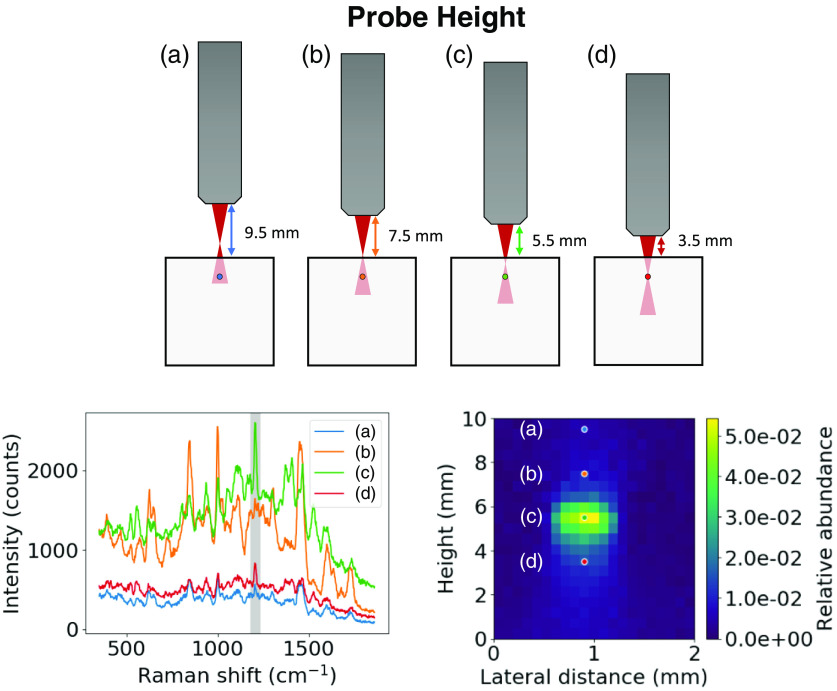
Schematic depiction of the phantom measurement setup at four axial positions (a) 9.5  mm, (b) 7.5 mm, (c) 5.5 mm, and (d) 3.5 mm along a single lateral position (top); representative spectra collected at the four axial positions (bottom left); and the resultant abundance map (bottom right). The shaded peak in the Raman spectra corresponds to the major peak of the SERS nanoparticle solution. The focal distance of the probe is 7.5 mm.

The 3D-printed phantom contained 1-mm diameter cylindrical channels at depths of 1 – 6 mm below the surface in a 76×50×20  mm (L×W×H) block. Each channel was filled with SERS nanoparticle solution and then centered below the probe before measurement. Raman spectra were collected point-by-point while translating the probe in the lateral and axial directions above the surface of the phantom. The laser power was set to 150 mW at the sample, resulting in an irradiance of $1.56\;W/{\mathrm{cm}}^{2}$ (the maximum permissible exposure for skin at 785 nm) using a 3.5-mm limiting aperture per the American National Standards Institute (ANSI) Z136.1 determination of MPE for skin.^23^ Scans were performed with the probe tip 0-10 mm above the surface of the phantom, in steps of 0.5 mm, and laterally across the channel, covering a distance of 2 mm in 0.1-mm steps. Figure 2 shows the measurement setup and how the collected Raman spectra relate to the abundance maps. SNR was calculated for each abundance map. The signal was taken as the mean value of a region of interest drawn over the area of high particle abundance, thresholded to 50% of the maximum. Background and noise were determined from the mean and standard deviation of the bottom ten rows of the first and last two columns in each map, respectively.

### 3.3 Data Processing

Python 2.7 was used for data analysis and visualization (Anaconda Python Distribution, Anaconda, Inc.; Austin, Texas). Raman spectra were baseline corrected using the Vancouver Raman Algorithm, which performs an iterative polynomial fitting.^24^ Spectral unmixing was performed using the non-negative least squares (NNLS) method within the PySptools package.^25^

## 4 Results and Discussion

An overview of the technical specifications for the three Raman systems is shown in Table 2. Device 1 is a portable USB spectrometer, which uses a Hamamatsu CCD detector in a Czerny-Turner configuration with a 101-mm focal length. Device 2 consists of a 300-mm focal length Czerny-Turner spectrograph from Acton and a back-illuminated Princeton Instruments (PI) CCD camera. Device 3 is a purpose-built transmission spectrograph from EmVision, which is configured with a back-illuminated, deep-depletion Andor CCD detector.

**Table 2:** Technical specifications of the three different Raman systems evaluated in this report.

	Device 1	Device 2	Device 3
Sensor	BI	BI	BI LDC-DD
Active px	1024×58	1340×400	2000×256
Pixel size	24 μm	20 μm	15 μm
Sensitivity	$27\;e^{-}/\mathrm{ct}$	$1\;e^{-}/\mathrm{ct}$	$1.5\;e^{-}/\mathrm{ct}$
QE at 800 nm	80%	∼72%	95%
f/#	4	3.9	2.2
Relative cost	$	$$$$	$$
Size (L×W×H)	182 mm × 110 mm × 47 mm (integrated CCD sensor)	368 mm × 254 mm × 211 mm (without camera)	280 mm × 246 mm × 142 mm (without camera)
Spectral range	Fixed grating: 599 to 984 nm	Motorized grating: 65 nm coverage	Fixed grating: 807 to 940 nm

BI = back illuminated, LDC = low dark current, DD = deep depletion.

### 4.1 Noise Characterization

**Fig. 3:**
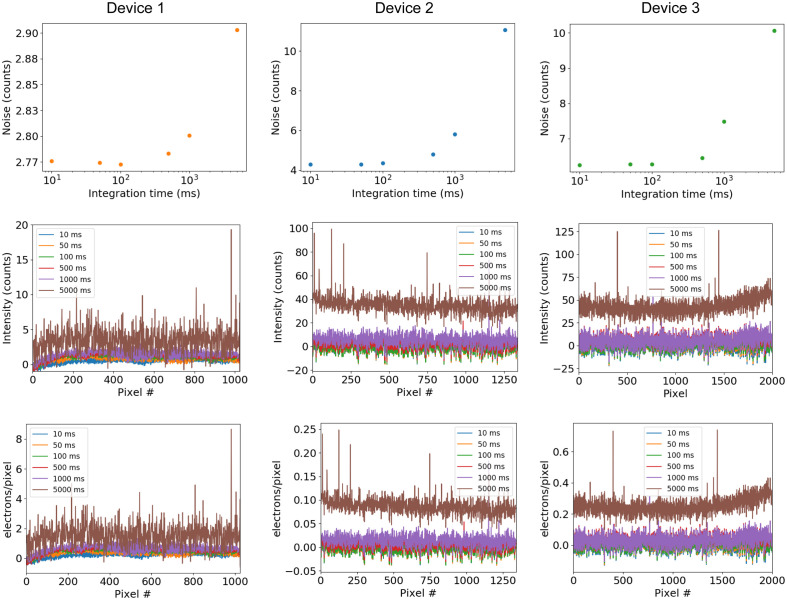
Dark noise as a function of integration time (top) and dark noise spectra from the three Raman systems at different integration times as specified. Each spectrum is the average of 100 acquisitions.

The noise characteristics of the three detectors were evaluated by recording dark spectra at different integration times (Fig. 3), similar to the method presented by Zonios.^21^ There are two forms of noise that originate from the CCD sensors – readout noise and dark noise. At short integration times (typically <1  s), readout noise dominates, while longer integration times allow for the dark current to accumulate and become the primary source of noise. Cooling of the sensor is often employed to reduce the effects of dark noise. All three Raman system detectors use thermoelectric cooling to minimize the dark current; Device 1 was operated at −20°C, while Devices 2 and 3 were operated at −60°C. It should be noted that decreasing sensor temperature will reduce quantum efficiency, so in cases with short integration times, it may be beneficial to identify the minimum amount of cooling needed where dark noise is no longer the limiting factor.

As shown in Fig. 3, all three systems are read noise limited below an integration time of approximately one second, where the dark noise remains relatively constant. Dark noise spectra are given in Fig. 3 to illustrate what this looks like in practice. At a five second integration time, the dark spectra of Devices 2 and 3 exhibit a marked increase in intensity, while the spectrum from Device 1 is slightly elevated but with higher variability. In terms of counts, Device 1 has lower noise than either Devices 2 or 3. This difference is due to having fewer vertical pixels for binning and the lower sensitivity of the Device 1 CCD sensor, where $27\;e^{-}$ are needed to generate a count as opposed to $1\;e^{-}$ and $1.5\;e^{-}$ with Device 2 and 3 CCDs, respectively. When converted to units of $e^{-}/\mathrm{px}$, we can see that Device 1 generates the highest dark current, as to be expected by the limited cooling capacity of the sensor. Device 2 generates about a third of the dark current of Device 3, which can be primarily attributed to the deep-depletion CCD of Device 3 that provides higher quantum efficiency in the NIR but also results in higher dark current.

**Fig. 4:**
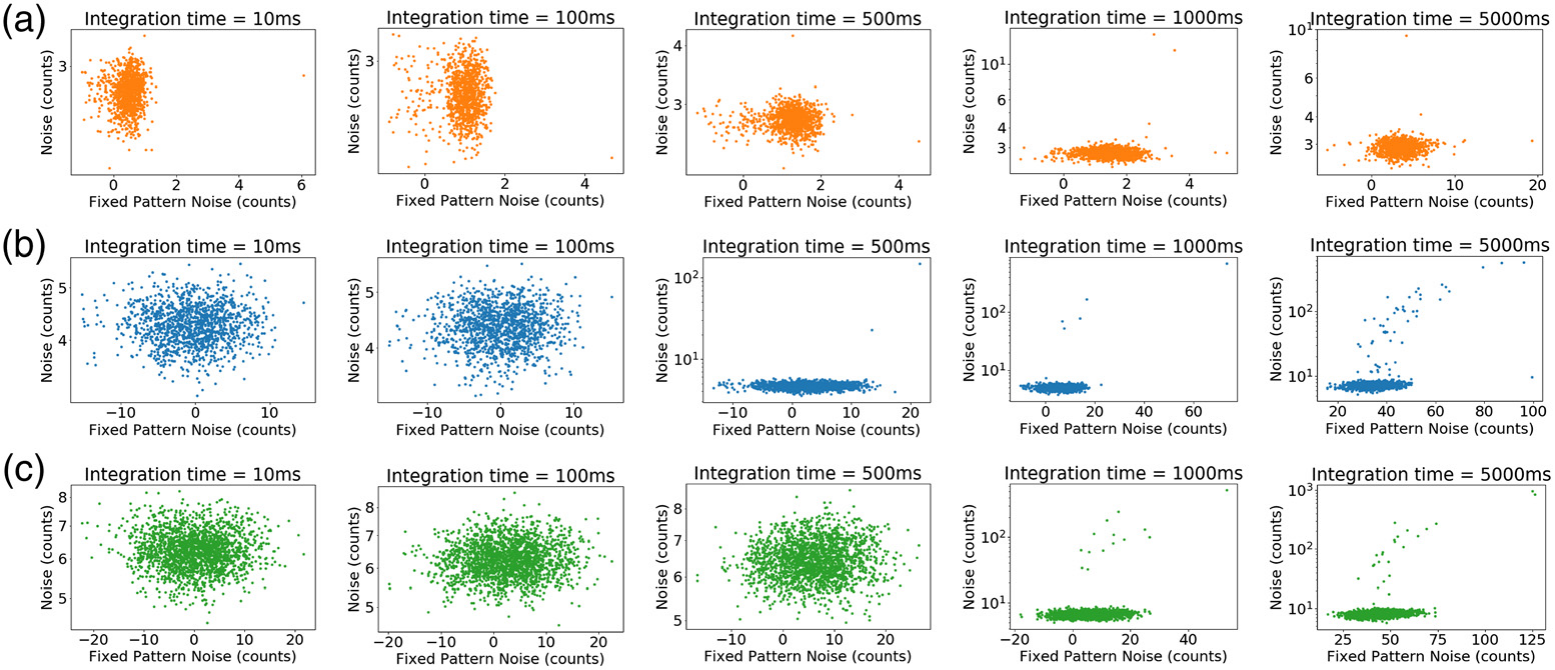
FPN of (a) Device 1, (b) Device 2, and (c) Device 3 at the specified integration times. For each pixel, the standard deviation (noise) is plotted on the y axis, with the mean (fixed pattern offset) on the x axis (n=100).

The spectra recorded with a five second integration time clearly show spike-like features resulting from fixed-pattern noise (FPN). The FPN is caused by differences in pixel responsivity across the image sensor. With increasing integration time, these differences are amplified and can interfere with measurements in light-starved applications. Figure 4 shows the noise measured at each pixel against the fixed-pattern offset for the Raman systems at various integration times. Devices 1 and 2 exhibit FPN at a 500-ms integration time, indicated by the change in clustering of points along the x axis. On Device 3, FPN is not evident until integration times of 1000 ms or longer. From these plots, it is evident that binning more vertical pixels will result in a higher amount of FPN. Devices 2 and 3 have a similar number of overall pixels, with Device 2 having about 1.5× the vertical pixels as Device 3.

### 4.2 Raman Shift Calibration

**Fig. 5:**
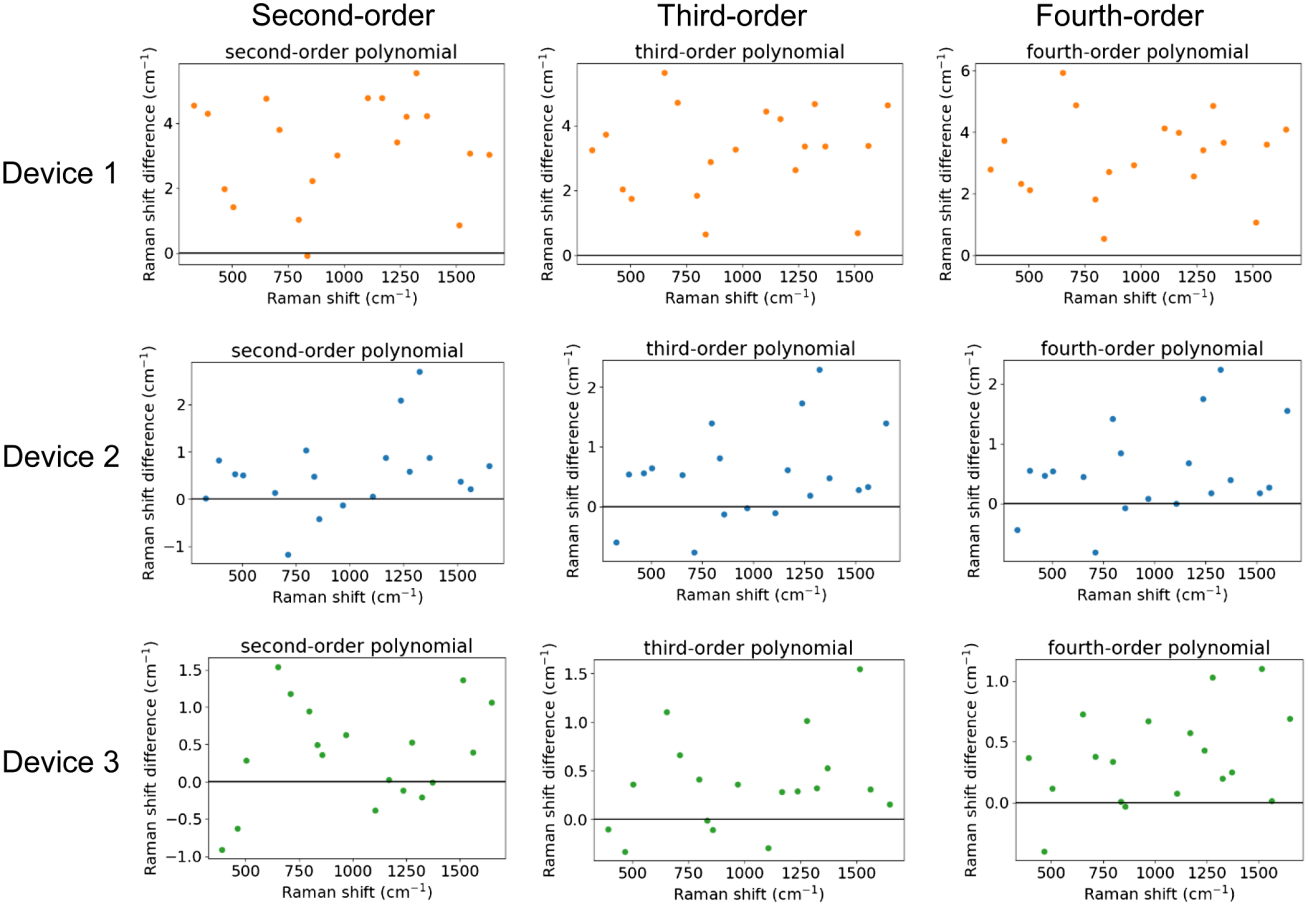
Error in acetaminophen Raman peak positions using different order polynomials for wavenumber calibration on the three Raman systems.

Acetaminophen was selected out of the eight Raman shift standards given in ASTM E1840 as it is widely available, inexpensive, easy to handle, and has also been used in a number of biomedical Raman studies.^5^^,^^26^^,^^27^ It contains the most peaks within the fingerprint region (17 points in the range 400 to $1800\;{\mathrm{cm}}^{-1}$) of all Raman shift standards in ASTM E1840, which is beneficial to obtaining a high-accuracy calibration. Raman spectra of acetaminophen were collected on each system and 19 peaks across the spectral range were used for calibration to pixel values.^18^ The measured peak pixel values were used for analysis without any fitting. For applications requiring higher accuracy, more advanced techniques involving peak fitting can be used.^28^ We compared the accuracy of three different polynomial interpolating functions for calibration of the wavenumber axis (Fig. 5). The root-mean-square error (RMSE) for each interpolating function is listed in Table 3. While a linear fit can be used to calibrate pixel values to wavelength, higher order polynomials must be used for Raman shift calibration due to the relative relationship between wavenumber and wavelength; although gratings disperse light linearly in wavelength, the dispersion in wavenumber is non-linear. Device 1 was shown to have the highest error in measured Raman shift, followed by Devices 2 and 3, respectively. The second-order polynomial was found to have the highest error of the three polynomials evaluated. Using a fourth-order polynomial resulted in a 2.5% reduction in error for Device 1, a 7.7% reduction in error for Device 2, and a 31.6% reduction in error for Device 3. The higher error of Device 1 may be explained by the fact that the Raman spectrum is being recorded from one end of the CCD sensor, where aberrations will be higher. It is also noted that the higher order polynomials provide little reduction in error for Devices 1 and 2, which have a Czerny–Turner configuration spectrograph, but a significant reduction in the error of the lens-based transmission spectrograph (Device 3). These results indicate that spectrometer design may influence the selection of an optimal calibration method.

**Table 3:** RMSE of the calibration interpolating functions in Fig. 5.

RMSE (${\mathrm{cm}}^{-1}$)	Second-order	Third-order	Fourth-order
Device 1	3.56	3.49	3.47
Device 2	0.98	0.92	0.91
Device 3	0.76	0.60	0.52

### 4.3 Relative Intensity Correction

**Fig. 6:**
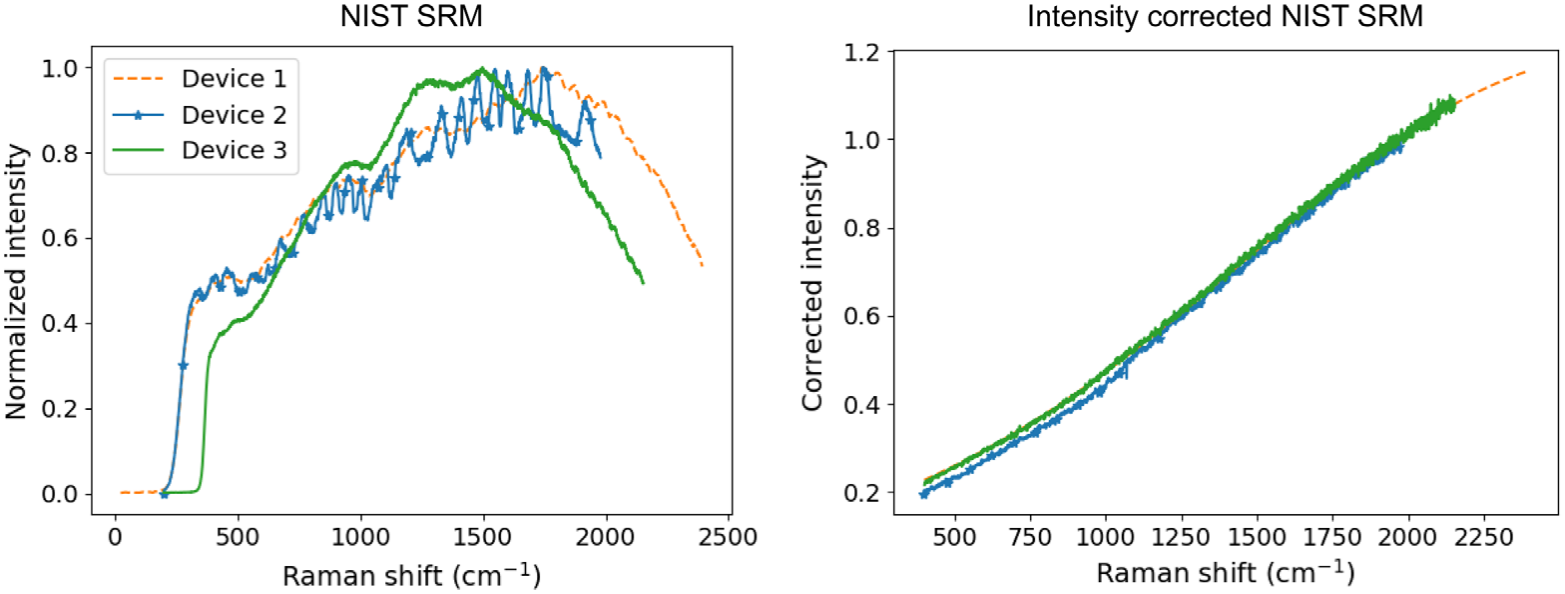
The measured luminescence of NIST Standard Reference Material 2241 (a) before and (b) after correction.

The differences in optical throughput and response of the Raman systems were corrected using NIST SRM 2241, as described in ASTM E2911.^19^ Figure 6 shows the variation in measured spectral shape between the three systems. The Device 3 spectrum cuts on at a longer wavenumber than the others due to the spectrometer’s integrated laser blocking filter. Interference fringes due to etaloning can be observed in the Device 2 spectrum. This is a known limitation of back-illuminated CCDs in the NIR range; however, more recent designs incorporate anti-fringing technology to suppress this effect. For example, Device 1 also uses a back-illuminated CCD. While some undulations can be seen in the far wavenumber region of the spectrum, they are nowhere near as severe or noticeable. A correction curve was created for each system using the relation between the measured luminescence spectrum to the certified polynomial model found in the NIST SRM 2241 Certificate. As shown in Fig. 6, the intensity-corrected luminescence spectra are all similar in shape, allowing for inter-device comparison of relative Raman peak intensities.

### 4.4 Spectral Resolution

**Fig. 7:**
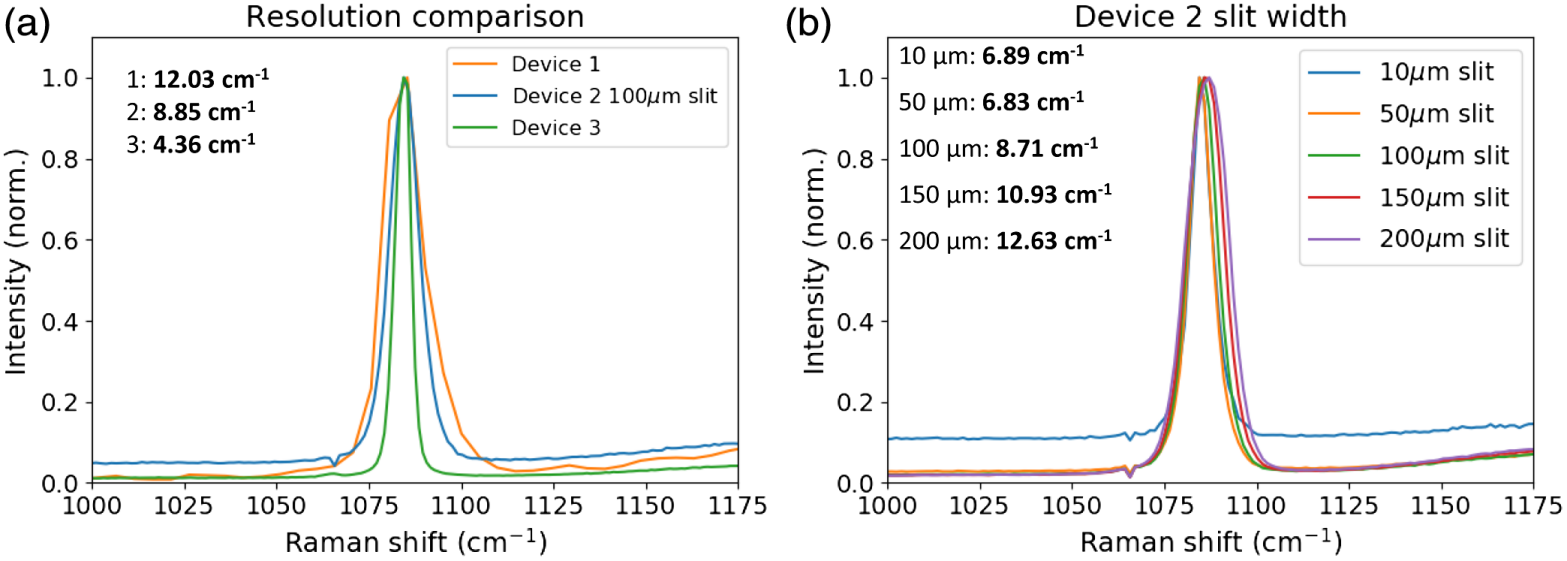
(a) Measured resolution of the Raman systems using the $1085\;{\mathrm{cm}}^{-1}$ peak of calcite. Device 1 and 3 have fixed slit widths of 50 and 100  μm, respectively, while Device 2 has a variable slit that was set to 100  μm. (b) Calcite spectra recorded with Device 2 at different slit width settings.

Resolution of the Raman systems was evaluated using the calcite method described in ASTM E2529. The measured full width at half height of the $1085\;{\mathrm{cm}}^{-1}$ ${\mathrm{CaCO}}_{3}$ Raman band is input to a calibration relation that was determined using a Fourier-Transform Raman instrument.^29^ Figure 7 (left) shows the spectra and calculated resolution of the three systems. Devices 1 and 3 have fixed, interchangeable slits at the spectrometer input. Device 1 was configured with a 50-μm slit and Device 3 with a 100-μm slit. Device 2 has a variable slit width that was set to 100  μm for this measurement.

Device 1 was found to have the lowest resolution ($12.03\;{\mathrm{cm}}^{-1}$) of the three evaluated, while Device 3 had the highest ($4.36\;{\mathrm{cm}}^{-1}$). Since the slit width of the Device 2 is variable, we evaluated its effect on the measured resolution. Spectra were collected using slit widths ranging from 10 to 200  μm, which are manually adjusted using the micrometer knob on the slit (Fig. 7, right panel). Reducing the slit width from 100 to 50  μm slightly improves the resolution, but a further reduction to 10  μm did not affect the measured resolution. This indicates that the resolution is limited by the dispersion of the grating at slit widths <50  μm. While reducing the slit width can improve resolution, it also reduces the amount of light entering the spectrometer, which may be problematic for measuring weak signals. This can be observed in the spectrum collected at a 10-μm slit width, where the peak-normalization results in an elevated baseline compared to spectra collected at larger slit widths.

### 4.5 System Sensitivity and Linearity

**Fig. 8:**
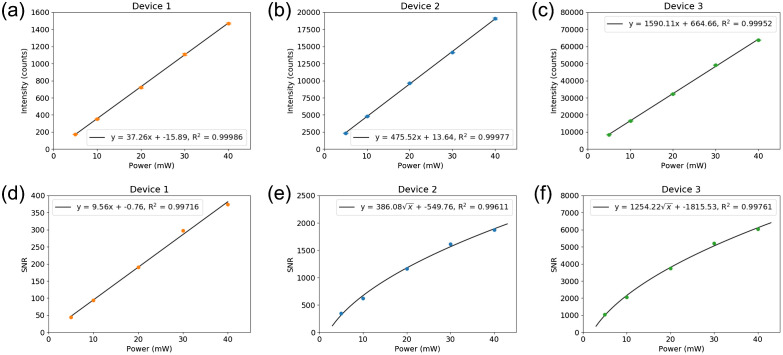
Comparison of Raman system sensitivity. The calcite $1085\;{\mathrm{cm}}^{-1}$ peak intensity was measured as a function of incident laser power with a one second integration time. Results are presented in units of intensity (a) – (c) and SNR (d) – (f). Error bars are ±1 standard deviation (n=3).

Another important aspect of Raman device performance is the sensitivity of the system. While detector specifications include a quantum efficiency curve and report sensitivity in terms of the number of electrons needed to generate a count, spectrometer throughput can significantly impact the amount of light reaching the detector. The conventional Czerny-Turner spectrograph design, which is used in Devices 1 and 2, has a F-number of ∼4 and uses a reflective grating. This high F-number results in a loss of collection efficiency when light is delivered to the spectrometer using a common 0.22 NA multimode fiber. In contrast, Device 3 has a lens-based transmission spectrograph with a F-number of 2.2, which can collect 100% of the light from a 0.22 NA fiber. Transmission gratings also offer higher efficiency (up to 90% absolute) compared to reflective gratings (∼70% maximum absolute efficiency), with lower wavelength and polarization dependence. To compare the sensitivity of the Raman systems, intensity of the calcite $1085\;{\mathrm{cm}}^{-1}$ peak was measured as a function of incident laser power (Fig. 8).

All three systems showed a high degree of linearity ($R^{2}>0.999$) in signal intensity with laser power, as expected based on the detector specification sheets. The slope of the intensity vs. laser power linear fits gives an estimate of system sensitivity. Device 1 had the lowest sensitivity with a slope of 37  counts/mW, Device 2 had a slope of 476  counts/mW, and Device 3 had the highest sensitivity with a slope of 1590  counts/mW. The high-throughput design of the transmission spectrograph in Device 3 shows an obvious advantage in sensitivity compared to the other two systems. This advantage would become even more apparent if the three systems were resolution matched. The results were also plotted in units of SNR versus laser power, shown in Figs. 8(d) – 8(f). Device 1 continued to show a linear trend with laser power; however, the SNR of Devices 2 and 3 were linear with the square root of laser power. This behavior is expected due to the increase in shot noise as laser power is increased. The lower sensitivity of Device 1 is unable to detect the slight increase in shot noise over the range of laser powers used.

### 4.6 Stray Light

**Fig. 9:**
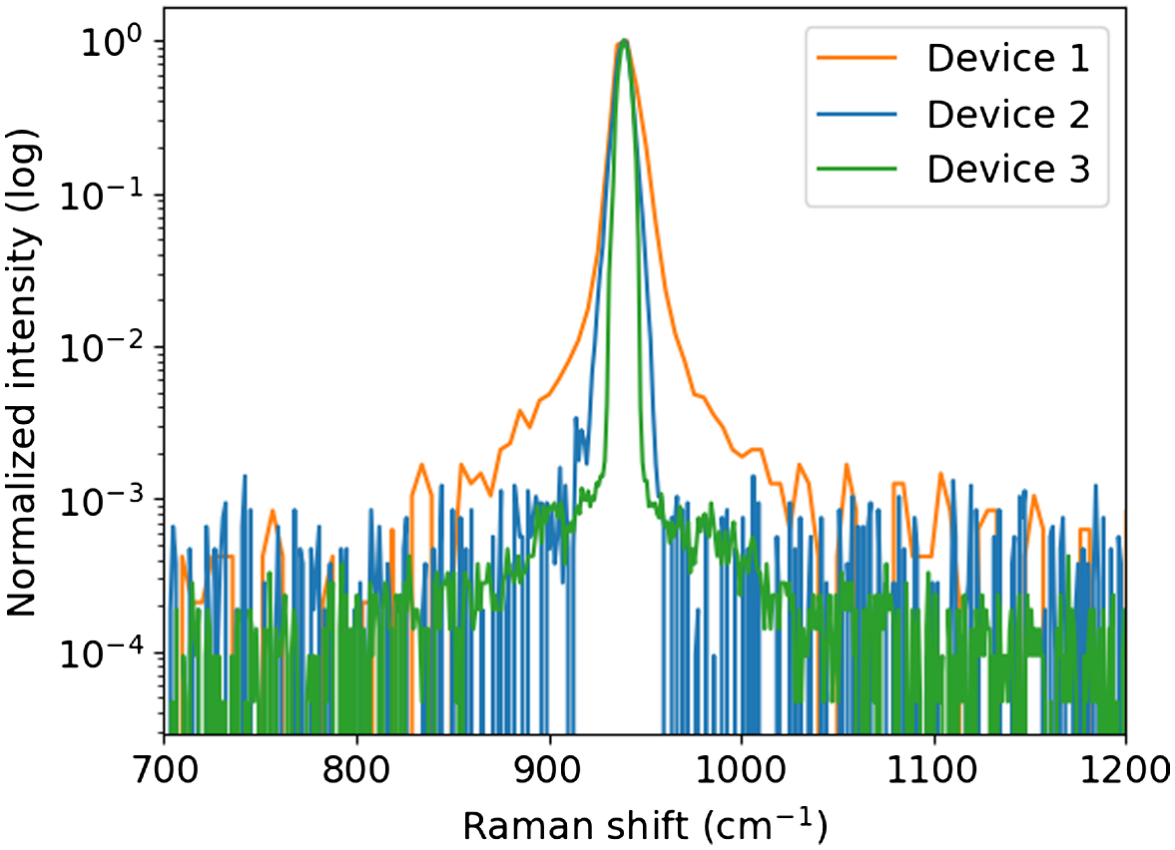
The measured spectrum of an 850-nm diode laser collected on each Raman system.

Stray light rejection is often specified in different ways by different manufacturers, so an empirical approach to its evaluation is recommended by ASTM E1683.^17^ In this case, we used the output of an 850-nm diode laser to compare spectral broadening effects between systems (Fig. 9). As the laser linewidth is narrower than the resolving power of the spectrographs, any differences in measured peak width can be attributed to stray light. Device 1 showed the greatest amount of broadening, which is not unexpected due to its compact design. Devices 2 and 3 exhibited less broadening, with Device 3 having the narrowest measured peak width.

### 4.7 Phantom Measurements

**Fig. 10:**
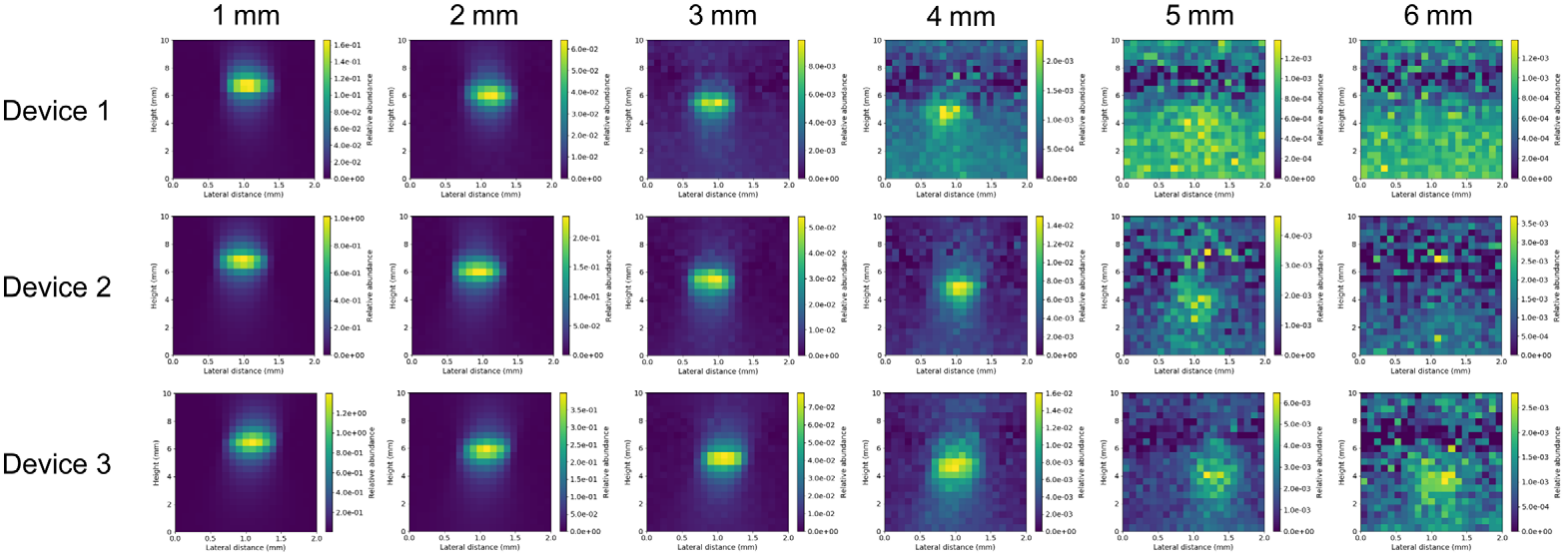
Abundance maps of SERS nanoparticle solution in channels at different depths below the surface of the phantom. It is noteworthy that the color scale varies between maps to aid in visualization. Each abundance map represents the axial position of the probe above the phantom from 0 to 10 mm and lateral position from 0 to 2 mm (i.e., each pixel represents 0.5 mm in the vertical direction and 0.1 mm in the horizontal direction).

While the testing implemented in the previous sections can provide important information regarding the basic performance specifications of Raman systems, they are not sufficient to indicate how a system would perform in practice for clinical applications. A tissue-mimicking phantom was implemented to compare the detectability of SERS particles at various depths using the three systems. We previously showed that our 3D-printed phantom can mimic the optical properties and Raman scattering of brain tissue.^14^ The phantom contained 1-mm diameter channels at depths of 1 to 6 mm below the surface. Using the 7.5-mm focal length probe, each channel was scanned in the axial (0- to 10-mm probe-to-surface distance or “height,” 0.5 mm steps) and lateral (0 to 2 mm, 0.1-mm steps) directions, with a Raman spectrum collected at each point (1 s integration time). Non-negative least squares (NNLS) spectral unmixing was used to extract the SERS particle signal contribution in the measured spectra to generate abundance maps (Fig. 10).

As channel depth increases, the probe must be placed closer to the phantom surface—thus positioning the focal point at a greater depth, to achieve the greatest particle signal. A banding effect is observed at around 7.5-mm height for all channel depths, becoming more prominent in the abundance maps of the deeper channels due to lower particle signal, and thus a reduction in the dynamic range of the color scale. In this region, Raman scattering of the phantom material dominates the measured spectrum as the probe is focused on the phantom surface. This results in the unmixing algorithm over-weighting the phantom material abundance, which presents as a dark band in the particle abundance map. The detectability of the systems follows the same trend found in the device sensitivity evaluation (Fig. 8; Device 3 > Device 2 > Device 1). To quantify this trend, contrast-to-noise ratio (CNR) measurements were performed on each of the abundance maps (Table 4). As shown in Fig. 10, it becomes difficult to distinguish the particle signal from the background below a CNR in the range of 1 to 2.

**Table 4:** Calculated CNR for the abundance maps shown in Fig. 10.

	1 mm	2 mm	3 mm	4 mm	5 mm	6 mm
Device 1	394	227	32.2	3.68	0.791	0.161
Device 2	434	210	53.4	19.6	4.69	1.02
Device 3	748	309	141	22.4	6.03	2.33

Qualitative observation of Fig. 10 indicates that Device 1 is able to detect the SERS particles to a depth of 4 mm, Device 2 can detect particles to 5 mm, while Device 3 can detect particles to 6 mm. This is in agreement with the calculated CNR, where the values above two are readily observed in the abundance maps. It is interesting to note how similar the three systems perform, especially for shallower channel depths, despite the significant differences in performance specifications that were identified in previous sections. One might expect the performance of Devices 2 and 3 to be much greater than Device 1 based on system sensitivity (Fig. 8). However, additional factors, such as probe design and the impact of turbid media must be taken into consideration when performing *in vivo* Raman measurements. Our results indicate that higher sensitivity and throughput of an instrument do not directly correlate to better performance in practice. This highlights the need for phantom-based test methods to assist in the characterization of Raman device performance under conditions simulating their intended use.

**Fig. 11:**
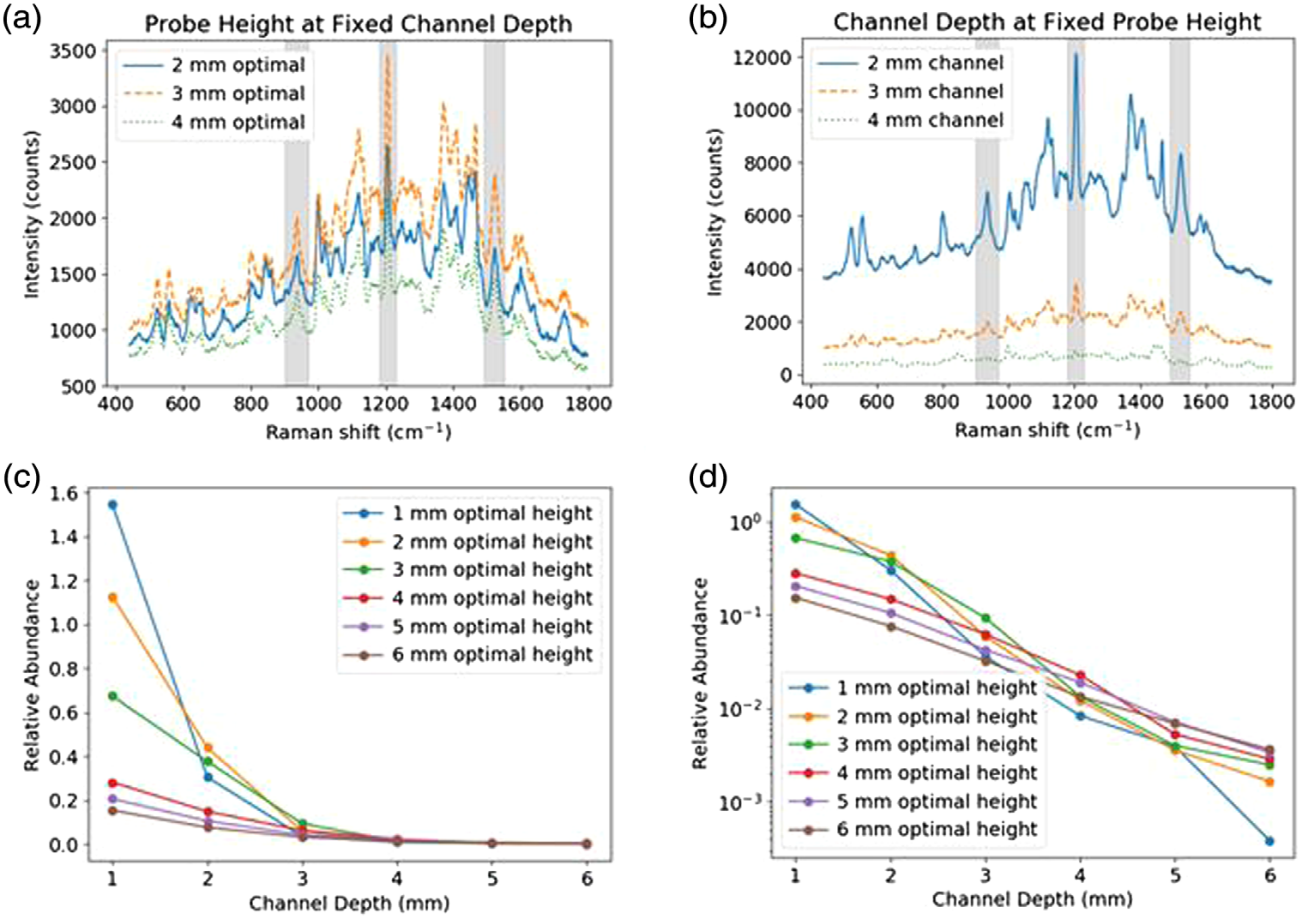
(a, b) Representative, non-background subtracted spectra collected with the (a) 3-mm deep channel, at the 2 to 4 mm channel depth optimal probe heights above the phantom surface, and (b) 3-mm optimal probe height, 5.5 mm above the phantom surface, for the 2 to 4 mm deep channels. The shaded regions in (a, b) correspond to major peaks of the SERS particles. (c) and (d) Relative abundance versus channel depth at the optimal probe height for each channel depth as indicated with a linear (c) and log (d) scaled y axis. The optimal probe heights for the 1-, 2-, 3-, 4-, 5-, and 6-mm channel depths are 6.5, 6.0, 5.5, 4.5, 4, and 3.5 mm, respectively.

We also studied the effect of illumination-collection geometry on depth selectivity in the turbid phantom. Device 3 was used for all measurements, with SERS particles located within the channels of the phantom. Figure 11(a) shows representative spectra collected at different probe heights with the 3-mm deep channel. These spectra follow the trend that could be inferred from Fig. 10, in that the highest particle signal (peaks shaded in gray) is obtained at the optimal probe height for the channel (2-mm deep channel: 6 mm; 3-mm deep channel: 5.5 mm; 4-mm deep channel: 4.5 mm), decreasing above and below this height. In Fig. 11(b), spectra collected at the 3-mm channel depth optimal probe height for 2 - 4 mm deep channels are given. While this probe height provides the highest particle signal for the 3-mm depth channel as shown in Fig. 11(a), higher particle signals are observed at shallower channel depths. As shown in Figs. 11(c), 11(d), for any fixed height value the probe always produces a monotonic decrease in particle abundance with channel depth. The data in Fig. 11(d) are the same as Fig. 11(c), but on a logarithmic y axis to aid in visualization of the optimal probe height at each channel depth. In general, changes in probe height produce variations in the slope of this decay, i.e., differences in depth-wise sensitivity, such that the maximum abundance for a specific channel depth depends on probe height; however, this does not result in an absolute increase in abundance compared to more superficial channels.

These results illustrate the ability of the 3D-printed phantom to be used in the assessment of illumination-collection design and resultant spatial sensitivity. This information may be useful for predicting the clinical performance of a specific probe, for example, by showing whether a probe is designed to provide optimal sensitivity to Raman scatterers with a known depth, such as a tissue layer where carcinogenesis tends to arise. Phantom-based methods are applicable not only to fiber probe Raman systems and may be useful in evaluating other optical delivery configurations (e.g., free-space, microscopy).^30^ Such methods can also be used to inform fiber-optic probe design, compare performance of different SERS nanoparticles, compare spectral processing methods, and investigate novel Raman techniques such as shifted-excitation Raman difference spectroscopy (SERDS).^14^^,^^30^^,^^31^ In the future it may be possible to include fluorophores into the phantom material to simulate tissue autofluorescence (e.g., from collagen, melanin) and study its impact on Raman device performance and background subtraction algorithms.^30^^,^^32^ Similar approaches have previously been implemented for performance testing of near-infrared fluorescence imaging devices.^33^

### 4.8 Summary of Experimental Results

Current standards for Raman devices identify a number of key performance specifications. Demonstrating consistency in these specifications will be important for any device that is produced for clinical use. Of note, Raman shift calibration is especially important when using laser diodes, where the exact wavelength is not known (typical center wavelength tolerance of ±0.5  nm), that have become ubiquitous in NIR Raman applications. Relative intensity correction is also necessary to compensate for any differences in the optical components from device-to-device. A clinical Raman device must be able to resolve the peaks of interest related to the biochemical composition of tissue (generally $∼5-10\;{\mathrm{cm}}^{-1}$ resolution is sufficient). While the noise, sensitivity, and stray light rejection are important characteristics that are necessary to ensure device consistency, additional complementary information is needed to evaluate the overall performance of different Raman devices for their intended use. Phantom-based test methods are also important in order to account for the impact of biological tissue turbidity and probe design. Such an approach can provide insight into the actual performance of a Raman device when used *in vivo*, which may not be apparent from the device specifications alone.

## 5 Conclusion

We reviewed the existing consensus standards related to Raman device performance and implemented recommended strategies on three different Raman systems. The three systems were shown to vary greatly in their resolution, sensitivity, and stray light rejection, with the trend of Device 3 > Device 2 > Device 1. However, their ability to detect a target at different depths in a tissue-mimicking phantom was surprisingly similar. These results indicate the need for phantom-based test methods for the standardized evaluation of Raman devices, as specifications alone are likely insufficient to predict *in vivo* performance. Such methods will enhance future efforts towards the clinical translation of Raman technology by aiding in regulatory evaluation, constancy testing, and ensuring manufacturing quality.

## Code, Data, and Materials Availability

The 3D-printed phantoms used in this study were made from commercially available materials. The authors can provide additional details upon request.
