# Evaluation of standardized performance test methods for biomedical Raman spectroscopy (Erratum)

**DOI:** 10.1117/1.JBO.27.7.079801

**Published:** 2022-07-21

**Authors:** Andrew M. Fales, Ilko K. Ilev, T. Joshua Pfefer

**Affiliations:** U.S. Food and Drug Administration, Center for Devices and Radiological Health, Silver Spring, Maryland, United States

## Abstract

The erratum corrects an error in Fig. 4 of the published article.

This article [*J. Biomed. Opt.* **27**(7), 074705 (2022) doi: https://doi.org/10.1117/1.JBO.27.7.074705 was originally published on 28 October 2022 with an error in Fig. 4. The axes were reversed in the original version:

**Figure:**
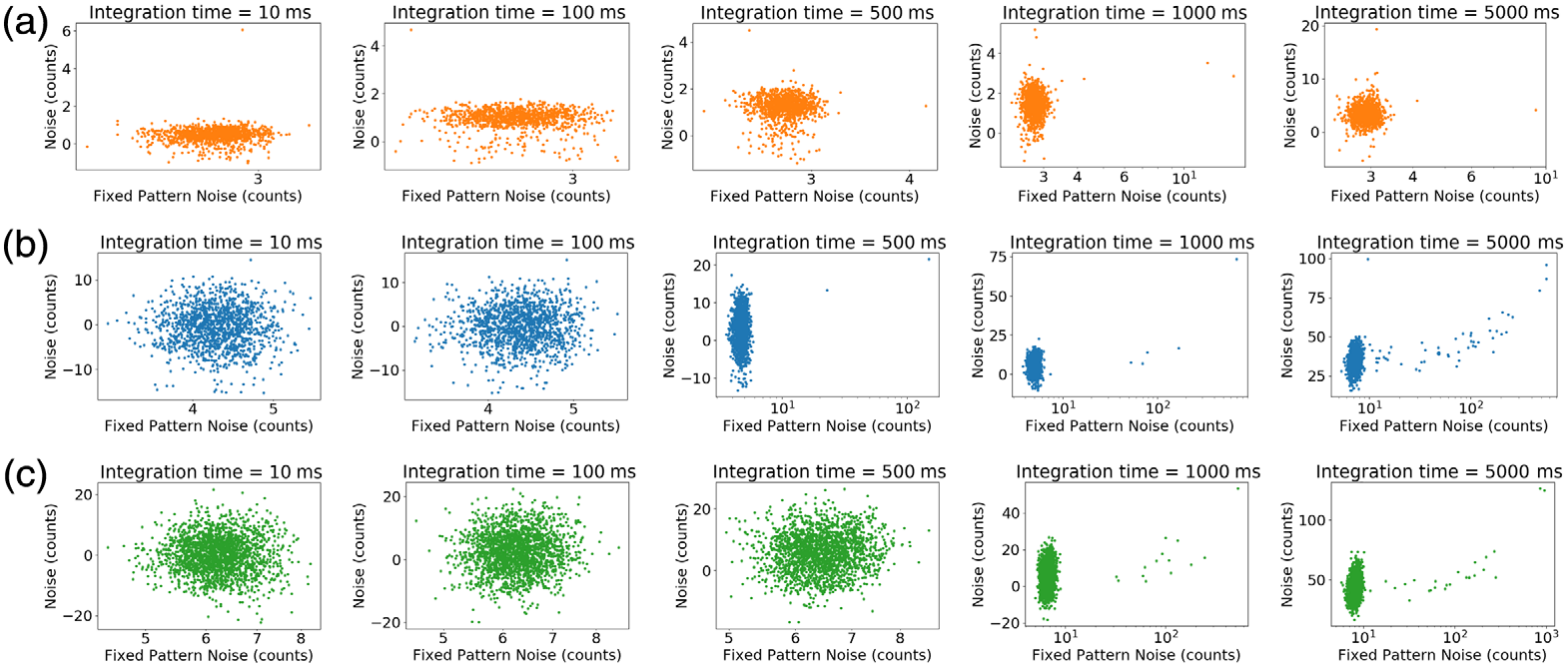


Corrected version:

**Figure:**
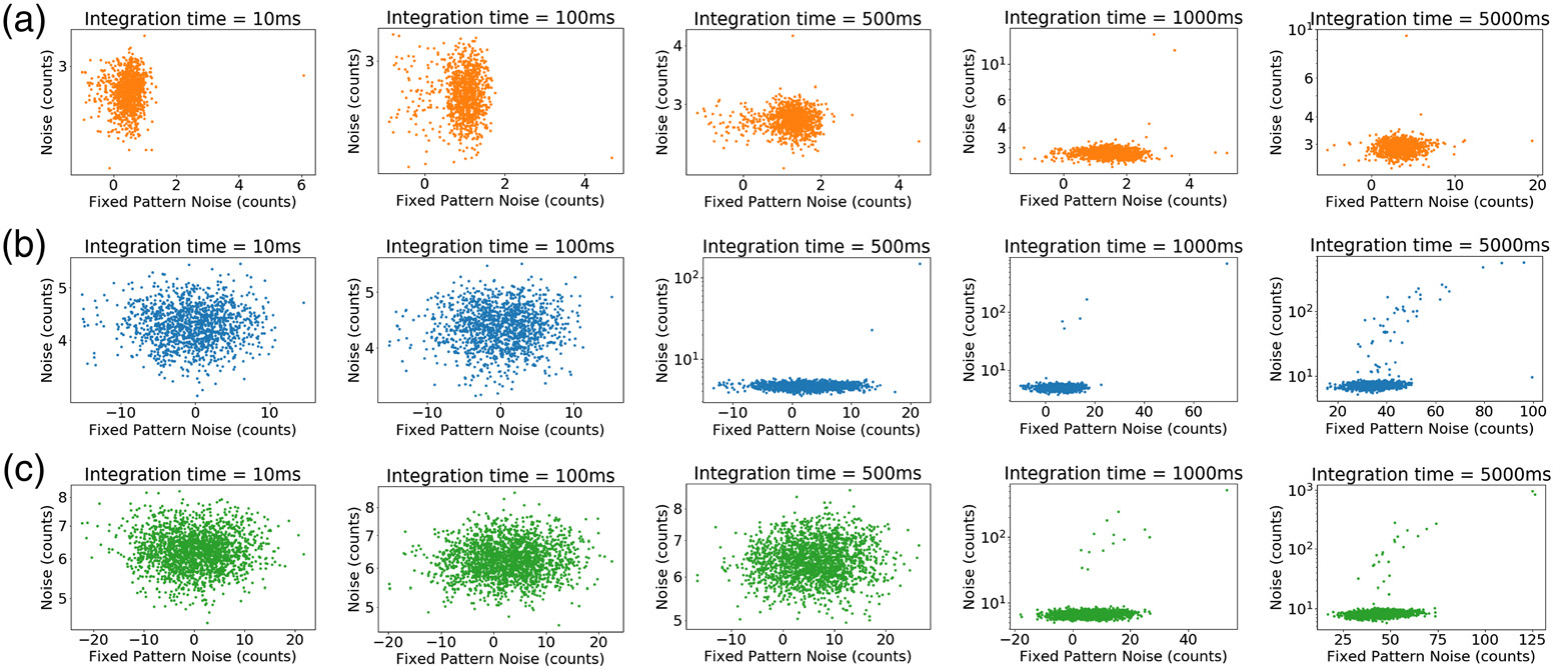


The error was corrected on 9 July 2022.

